# Correlation between quantitative traits and correlation between corresponding LOD scores: detection of pleiotropic effects

**DOI:** 10.1186/1471-2156-4-S1-S60

**Published:** 2003-12-31

**Authors:** Ayse Ulgen, Zhihua Han, Wentian Li

**Affiliations:** 1 G.H. Sergievsky Center, Columbia University, New York, New York 10032, USA; 2 Laboratory of Biochemical Genetics and Metabolism, The Rockefeller University, New York, New York 10021, USA; 3 The Robert S Boas Center for Genomics and Human Genetics, North Shore LIJ Research Institute, Manhasset, New York 11030, USA

## Abstract

**Background:**

We address the question of whether statistical correlations among quantitative traits lead to correlation of linkage results of these traits. Five measured quantitative traits (total cholesterol, fasting glucose, HDL cholesterol, blood pressure, and triglycerides), and one derived quantitative trait (total cholesterol divided by the HDL cholesterol) are used for phenotype correlation studies. Four of them are used for linkage analysis.

**Results:**

We show that although correlation among phenotypes partially reflects the correlation among linkage analysis results, the LOD-score correlations are on average low. The most significant peaks found by using different traits do not often overlap.

**Conclusion:**

Studying covariances at specific locations in LOD scores may provide clues for further bivariate linkage analyses.

## Background

If the same gene (pleiotropy) caused two quantitative traits, linkage analyses of these two traits would lead to a peak at the same region, and there would therefore be a statistical correlation of two sets of LOD scores in a specific region. On the other hand, if different genes caused two traits, no correlation is expected at the LOD score level unless there are tightly linked loci influencing both traits. In the case of pleiotropy, there should be a correlation between the two traits caused by the same gene. If two traits are highly correlated, the corresponding LOD scores from the linkage analysis would also be expected to be highly correlated, and it may therefore not be necessary to carry out linkage analysis twice. If the correlation between two traits were perfect then the correlation in LOD scores would also be perfect. Here we argue that any less-than-perfect correlation between the two traits may lead to quite different linkage analysis results, and that linkage analysis is therefore necessary for both traits.

The Framingham data [1] provides a chance to study this issue because measurements of many quantitative traits are available. In addition to some environmental factors, physical measurements (weight and height), and covariates (sex, age), there is information on five quantitative traits: total cholesterol (TC), fasting glucose (GLU), high density lipoprotein cholesterol (HDL), systolic blood pressure (BLP), and triglycerides (TG), which are largely independent of TC. We have added one more derived quantitative trait called cholesterol ratio [2], the ratio between TC and HDL (CR = TC/HDL). Any one of these quantitative traits can be used for a linkage analysis. The question we address is whether any statistical correlation present in the traits is reflected by correlations in linkage analysis results (even though we do not form the question in a hypothesis-testing framework, a null hypothesis can be tested, namely that the correlation coefficient between two traits is equal to that between two sets of LOD scores).

## Methods

### Data pre-processing (Cohort 1 and Cohort 2 difference)

The Cohort 1 and Cohort 2 files contain trait information for the older and younger generations, respectively, in the Framingham Heart Study. There is a huge difference in the amount of missing data between the two files. In Cohort 1, measurements were taken 21 times, though for some traits they were only measured a few times (e.g. three times for TG). In Cohort 2, measurements were taken five times and there are rarely missing data. For our analysis, for simplicity as well as for the purpose of removing certain environmental effects, we do not study the time sequence of these measurements, so the average of each trait is used.

### Data pre-processing (logarithm transformation of TG)

It is well known that TG fluctuates wildly. Even measured on the same person, TG value may change during a day and depends on whether one eats or not. The distribution of TG is highly skewed. To make the distribution more Gaussian-like, we apply a logarithm transformation (log(TG)).

### Correlation between traits

Pair-wise Pearson's correlation coefficient was calculated between six traits and the age (all averaged over the study period): TC, GLU, HDL, BLP, TG, and CR. For Cohort 1, one or a few trait values may not be available for some people. These persons are ignored in the corresponding correlation calculation. We also carried out a hierarchical cluster analysis of the six traits, using the Euclidean distance and average linkage. The traits BLP and GLU comprise one branch, which is separated from other branches and traits.

### Sex and age correction of quantitative traits

The male vs. female difference of a particular trait can be tested by an analysis of variance (ANOVA). Note that ANOVA for two categories is equivalent to a t-test. If the correlation between the age variable and another trait is significant, there is also an age effect on that trait. Such correction analysis is carried out by two separate, gender-specific, regressions:

*y*_*g *_= *c*_0,*g *_+ *c*_1,g _* *AGE*,     *g *= {*f*, *m*}.

### Quantitative trait linkage analysis

The computer program MERLIN [3] is used for the linkage analysis of quantitative traits. We use a single-marker variance component linkage analysis [4]. All pedigrees with larger than 20 "bit" value (a measure of the pedigree complexity) are split into sub-pedigrees (see the next subsection).

### Pedigree pre-processing for linkage analysis

Because of the limitation on the pedigree size when running MERLIN, we manually removed all untyped individuals who were deletable (i.e., they did not link two typed individuals). Large pedigrees were also split into two or more sub-pedigrees so that all had "bit" value less than 20 (before splitting, the largest "bit" values include 90 (ped 26526), 55 (ped 24619), 39 (ped 26671), 38 (ped 27992), 37 (ped 31116), etc. A total of 31 pedigrees were split into smaller pedigrees. After simplifying the pedigrees, the number of individuals was reduced to 4095 from the original number of 4692. A program RECODE [GR Abecasis, personal communication, 2002] was used to relabel ("downcode") allele values so that they started from 1.

### Correlation between LOD scores

The Pearson correlation coefficient is calculated for two sets of LOD scores obtained for the two traits. Each set of LOD scores consist of LOD scores on 398 markers,averaged over all families (LOD_i_, i = 1,2,...,398). Besides the correlation coefficients, scatter plots of a pair of LOD score sets are provided in order to discern any "outliers" (markers that behave very differently from the rest of markers).

## Results

### Correlation among traits

Table 1 shows the correlation coefficients among six traits (and age). Results for Cohort 2 and Cohort 1 are listed separately. We have the following observations: 1) HDL is negatively correlated with all other variables. We expected this result because HDL transports excess TG and cholesterols out of the bloodstream. 2) Generally speaking, the age effect on these traits (except HDL) is positive. 3) The derived quantity, cholesterol ratio (CR = TC/HDL), is highly correlated with the log(TG) variable even though CR is not derived from TG.

### Gender-specific effect on quantitative traits

Table 2 shows the gender-specific means of traits and the ANOVA test (or t-test) result for Cohort 2 and Cohort 1 separately. Since the measurements carried out for Cohort 2 are more complete, we rely more on this data set. Table 2 shows that there is a significant difference of GLU, HDL, BLP, log(TG), and CR between males and females. These results are not completely reproducible for the Cohort 1 data set, which is less reliable.

### Linkage analysis results of quantitative traits

**Figure 1:**
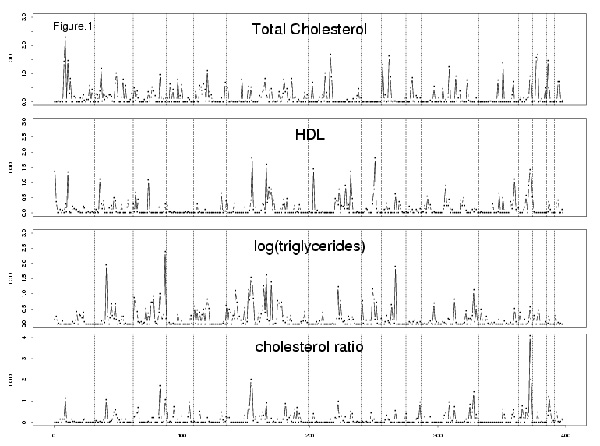
**LOD scores obtained from MERLIN for four quantitative traits (TC, HDL, log(TG), and CR) **Vertical lines partition markers on different chromosomes.

Cluster analysis shows that the two traits, BLP and GLU, are on a separate branch from the other four traits. For this reason, we decided to focus on the four more closely related traits, TC, HDL, log(TG), and CR, for linkage analysis. The LOD scores obtained from single-marker variance component linkage analysis [4] are shown in Figure 1 for these four quantitative traits. The vertical dashed lines partition markers in different chromosomes. The range of the y-axis for the last plot is larger (to 4) than the rest to accommodate the high peak for the marker on chromosome 19 (the marker Mfd232). MERLIN runs for quantitative trait linkage analyses (see  for details) were also carried out, but the results were not consistent with the variance-component linkage analysis runs and are not shown here.

**Figure 2:**
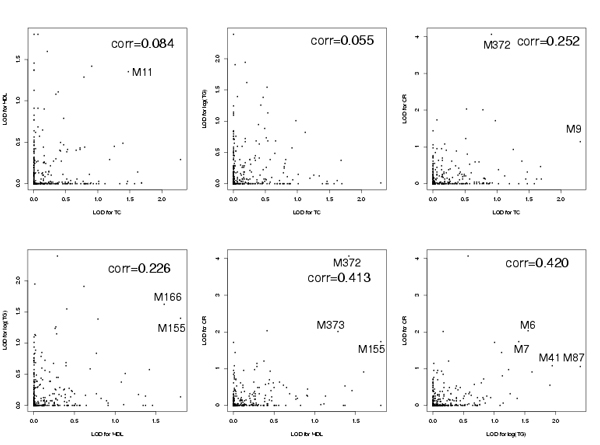
**LOD scores obtained from MERLIN for four traits as paired between any two traits (TC vs. HDL, TC vs. log (TG), TC vs. CR, HDL vs. log (TG), HDL vs. CR, and log (TG) vs. CR) **Each point represents a marker whose two LOD scores from the two traits are the x and the y coordinate value.

Figure 2 shows the LOD score of one trait versus that of another trait (for six trait-pairs among four traits). Each point in Figure 2 represents a marker. Correlation coefficients, which measure the average correlation of two LODs of all markers, are listed in Table 3. The strongest correlation is between HDL and CR (correlation coefficient is 0.413) and between log(TG) and CR (correlation is 0.420). Many points in Figure 2 gather around the origin, which are markers of no linkage signal for either trait. There are also many points that are near two axes-these have different linkage signals (some could be false positive)-between two traits. Outliers, which are points far away from the origin, are the most interesting markers because they indicate linkage signals for both traits. It can be seen that the presence of these outliers does increase the correlation coefficient value. Although Figure 2 does not contain information on which markers are nearby, the number of markers in this data set is sparse enough that we can consider them to be unlinked.

## Conclusions

It is clear from Figure 1 that peaks in linkage analysis of one quantitative trait do not lead to peaks in such analysis of another trait, even though the two traits might be somewhat correlated (as indicated by the correlation coefficients between traits in Table 1). The visual impression from Figure 1 also does not seem to match the correlation coefficients among these LOD scores (as shown in Table 3). Figure 2 provides further evidence that it is rare to have outliers-markers that exhibit relatively higher LOD scores for both traits. This implies that a less-than-perfect statistical correlation between two traits does not mean that *for each peak in LOD score for linkage analysis of one trait, there is also a matching peak in the linkage analysis of another*. The example of perfect correlation (i.e., trait 2 is a copy of trait 1) does not generalize to situations of less than perfect correlations. This observation has direct practical implication on linkage analyses of a few correlated quantitative traits, because the presence of one linkage signal for one trait may not lead to a linkage signal for another related trait (or, pleiotropic effect may not be detected from the trait correlation). The most striking example is the marker Mfd232 on chromosome 19: even though CR is derived from TC and HDL, the linkage signal for CR at this marker is much stronger than those for the TC or the HDL trait alone. Finally, we note that there is a possibility that two LOD score peaks at the same location may be caused by two closely linked genes instead of the same gene. In other words, we may not be able to distinguish the situation of pleiotropy and the situation of two linked genes. For all practical considerations, such distinction is minor.

## Figures and Tables

**Table 1:** Correlation coefficients among six traits (and the age).

Cohort 2	TC	GLU	HDL	BLP	Log (TG)	CR
Age	0.385^A^	0.257^A^	-0.0008	0.472^A^	0.299^A^	0.227^A^
TC	1	0.156^A^	-0.024	0.283^A^	0.450^A^	0.576^A^
GLU		1	-0.227^A^	0.359^A^	0.350^A^	0.284
HDL			1	-0.150	-0.546^A^	-0.783^A^
BLP				1	0.348^A^	0.285^A^
Log(TG)					1	0.729^A^
CR						1

Cohort 1						

Age	0.074	0.093^A^	0.0029	0.281^A^	-0.026	0.020
TC	1	0.013	-0.0066	0.137^A^	0.280^A^	0.479^A^
GLU		1	-0.159^A^	0.165^A^	0.160^A^	0.127^A^
HDL			1	-0.068	-0.451	-0.813^A^
BLP				1	0.165^A^	0.128^A^
Log(TG)					1	0.561^A^
CR						1

^A ^Significant (*p *< 0.01) result for testing zero correlation.

**Table 2:** Sex-specific means of seven variables and ANOVA test result (for Cohort 1 and Cohort 2 separately) and the linear regression result for six variables over the age (for data set combining Cohort 1 and Cohort 2).

	**Cohort 2**			**Cohort 1**			**Cohort 1 + 2 Regression on Age**			
	
	Male Mean	Female Mean	*p*-value	Male Mean	Female Mean	*p*-value	Male		Female	
							
							c_0_	c_1_	c_0_	c_1_
Age	42.35	43.50	0.0273	56.83	57.57	0.067				
TC	200.76	198.62	0.199	221.23	229.40	**4.4 × 10^-5^**	164.341	0.930	128.827	1.675
GLU	99.84	95.53	**5.6 × 10^-7^**	90.48	90.45	0.97	97.932	-0.022	86.019	0.148
HDL	43.95	55.07	**0**	43.86	53.56	**0**	45.752	-0.038	56.101	-0.0328
BLP	125.16	118.87	**0**	135.79	136.80	0.32	102.791	0.554	76.183	1.017
Log(TG)	4.72	4.47	**0**	4.79	4.67	**0.00057**	4.312	0.009	3.851	0.014
CR	4.81	3.79	**0**	5.44	4.54	**0**	3.552	0.031	2.239	0.037

**Table 3:** Correlation coefficients of four sets of LOD scores obtained from linkage analysis on TC, HDL, log (TG), and CR.

	**HDL**	**Log (TG)**	**CR**
TC	0.084	0.055	0.252
HDL	1	0.226	0.413
Log(TG)		1	0.420
CR			1
